# Elevated Levels of Soluble Axl (sAxl) Regulates Key Angiogenic Molecules to Induce Placental Endothelial Dysfunction and a Preeclampsia-Like Phenotype

**DOI:** 10.3389/fphys.2021.619137

**Published:** 2021-07-13

**Authors:** Shunping Gui, Shengping Zhou, Min Liu, Yanping Zhang, Linbo Gao, Tao Wang, Rong Zhou

**Affiliations:** ^1^Department of Obstetrics and Gynecology, Center for Translational Medicine, Key Laboratory of Birth Defects and Related Diseases of Women and Children (Sichuan University), Ministry of Education, West China Second University Hospital, Sichuan University, Chengdu, China; ^2^Center for Translational Medicine, Key Laboratory of Birth Defects and Related Diseases of Women and Children (Sichuan University), Ministry of Education, Department of Obstetrics and Gynecology, West China Second University Hospital, Sichuan University, Chengdu, China

**Keywords:** preeclampsia, endothelial dysfunction, placenta, sAxl, rat

## Abstract

Preeclampsia (PE), a severe pregnancy-specific syndrome, is characterized by impaired placental angiogenesis. Although the pathogenesis of this condition remains largely unclear, vascular systemic endothelial injury is thought to be the common contributing factor. Soluble Axl (sAxl), a biomarker of endothelial dysfunction, is known to be abnormally increased in a variety of diseases associated with vascular injury. In a previous study, we found that the plasma levels of sAxl were significantly higher in PE with severe features (sPE) than in pregnant women who did not have PE. The current study aimed to further explore the potential role of sAxl in vascular injury in patients with sPE. We found that the upregulation of sAxl in maternal plasma was positively correlated with the plasma levels of sFlt-1 and negatively correlated with placental NO synthase (eNOS) in women with sPE. Furthermore, elevated levels of sAxl suppressed proliferation and endothelial tube formation and promoted cytotoxicity in human umbilical vein endothelial cells (HUVECs) through the downregulation of p-Akt, p-p70S6K, p-mTOR, and Grb2. Subsequently, we established a pregnant rat model with PE-like characteristics by injecting pregnant rats with an adenovirus expressing sAxl. These rats exhibited a typical PE-like phenotype, including increased blood pressure, proteinuria, and fetal growth restriction, along with abnormal placental and fetal renal morphology. In conclusion, our study demonstrated the role of sAxl in systemic vascular injury through the regulation of the expression of key molecules of angiogenesis and described its potential contribution to the development of sPE.

## Introduction

Preeclampsia (PE), as one of the main causes of maternal and perinatal mortality, represents a significant threat to the health of both the mother and the fetus (Mol et al., 2016). PE is clinically described as the occurrence of high blood pressure after the 20th week of gestation, which can progress to multiorgan dysfunction, including hepatic, renal, and cerebral diseases if the fetus and the placenta are not delivered (Agius et al., 2018). When hypertension is severe (≥160/110 mmHg), or if there is evidence of maternal organ and system dysfunction, then this condition is referred to as PE with severe features (sPE) and represents a serious threat to maternal and fetal health and pregnancy outcome (Burwick et al., 2018). Furthermore, it has been reported that there are positive associations between hypertensive disorders in pregnancy and cardiovascular risk factors after pregnancy, which may subsequently lead to end-stage renal disease (Melchiorre et al., 2011; Dai et al., 2018). Although the causes of PE remain ill-defined, vascular systemic endothelial injury is thought to be the common factor that contributes to its development.

The two-stage theory is widely recognized by most researchers, including abnormal placentation and the development of maternal syndrome; the research has also shown that some pathological changes play an important role in both stages (Bujold and Hyett, 2019). Placental remodeling disorders associated with vascular dysfunction factors can lead to ischemia and hypoxia, while placental ischemia/hypoxia causes the release of circulating endothelial factors that target the blood vessels (Yu et al., 2018). Studies suggest that an imbalance of circulatory angiogenic and anti-angiogenic factors plays a pathological role in the etiology of maternal syndromes, such as high serum levels of vascular cell adhesion molecule-1 (VCAM-1) and soluble fms-like tyrosine kinase-1 (sFlt-1) with low expression levels of placental growth factor (PlGF) (Chaiworapongsa et al., 2002; Levine et al., 2004). In pregnant women with PE, an excess of sFlt-1 is secreted by the placenta, which then binds to local and circulating vascular endothelial growth factor (VEGF) and PlGF, thus resulting in the inhibition of VEGF and PlGF signaling in the vasculature (Phipps et al., 2019). This inhibition state subsequently impacts endothelial cell dysfunction, including the reduced production of nitric oxide (NO), prostacyclin, and the release of procoagulant proteins. A previous study reports that injecting sFlt-1 adenovirus into pregnant mice, as an animal model of PE, can increase blood pressure, proteinuria, and glomerular endothelial hyperplasia (Burke et al., 2016). Moreover, the anti-angiogenic protein further reduced the activity of endothelial NO synthase (eNOS), thus resulting in reduced NO availability and increased vascular permeability, which leads to systemic vascular dysfunction (Venkatesha et al., 2006).

Axl is a member of the TAM family of receptor tyrosine kinases and is widely expressed in many cell types, but is commonly found in the vasculature (O’Bryan et al., 1991). An extracellular segment of vascular endothelial Axl can be released by proteolytic cleavage through a wide variety of metalloproteinases, which releases an 80-kDa soluble protein (sAxl) into the blood circulation (Thorp et al., 2011). Growth arrest-specific 6 (Gas6) is the ligand for all TAM family members, including Axl (Wu et al., 2017). Besides, Axl has been shown to be necessary for *in vivo* angiogenesis and tumor development in mouse models (Holland et al., 2005). Moreover, the silencing of Axl is known to impair angiogenesis and cellular responses intrinsic to angiogenesis (Ruan and Kazlauskas, 2012). The inhibition of Axl impairs tumor cell-induced angiogenesis (Tanaka and Siemann, 2019, 2020). It is also known that Gas6/Axl regulates migration and anti-apoptosis of vascular smooth muscle cells *via* the PI3K/AKT/mTOR pathways. A previous study reports hyperglycemia-induced human endothelial dysfunction *via* an increase in intercellular adhesion molecule-1 (ICAM-1), VCAM-1 expression, and reduced Gas6/Axl expression (Lee et al., 2014). A further study also confirms that excessive sAxl can bind to Gas6 to reduce free Gas6 levels in blood circulation, thus inhibiting Gas6/Axl-mediated signaling pathway, and sAxl is considered to represent a biomarker of endothelial dysfunction (Batlle et al., 2016). Our previous study found that plasma sAxl was significantly higher in patients with sPE than pregnant women without PE (Liu et al., 2014). Thus, we speculate that a high level of sAxl in maternal plasma may cause vascular endothelial cell damage by inhibiting Gas6 and the downstream signaling pathway, thus leading to abnormal angiogenesis and the induction of sPE.

In this study, we carried out *in vitro* and *in vivo* experiments to investigate whether the circulating levels of sAxl in maternal plasma are positively correlated with the clinical characteristic of sPE; maternal circulating levels of sAxl might induce placental endothelial dysfunction and pathophysiology of sPE through the Gas6/Axl/AKT/mTOR pathways and the abnormal levels of key angiogenic molecules. We then established a rat model with high levels of sAxl by injecting pregnant rats with an adenovirus expressing sAxl; these model rats exhibited placental endothelial dysfunction and a PE-like phenotype.

## Materials and Methods

According to the Transparency and Openness Promotion Guidelines raised by the journal, the data that support the findings are available from the corresponding author upon reasonable request. This study conforms with standard biosecurity and institutional safety procedures.

### Plasma and Tissue Specimens

This study was approved by the Medical Ethics Committee of Sichuan University, and all subjects received written informed consent. This study was approved by the Institutional Ethics Committee of West China Second University Hospital. Sixty pregnant women who had been referred to the obstetrics unit of West China Second University Hospital from April 2011 to December 2012 were included. SPE was defined according to the International Society for the Study of Hypertension in Pregnancy (ISSHP) (James et al., 2013). SPE was characterized by hypertension (systolic blood pressure ≥ 160 mmHg and diastolic blood pressure ≥ 110 mmHg) after weeks of 20 gestation and 24-h proteinuria ≥ 2.0 g or other severe symptoms. In total, we enrolled 30 pregnant women with sPE, along with 30 premature healthy control subjects. All pregnant women underwent routine ultrasound measurements during the first trimester. In the third trimester, 5 ml of blood was extracted before therapeutic intravenous administration of magnesium sulfate; the blood was collected in a sterile EDTA-containing vacutainer tube and subsequently centrifuged at 1,600 rpm and 4°C for 10 min to allow the separation of plasma. Placental tissues were collected as described previously (Gui et al., 2014). All samples were stored at −80°C for further use.

### Enzyme-Linked Immunosorbent Assay (ELISA)

We quantified the levels of sAxl (Cat#DAXL00), Gas6 (Cat#DY885B), and sFlt-1 (Cat#DVR100C) in the plasma samples with appropriate human ELISA kits (USCN KIT Inc., R&D) following the manufacturer’s instructions. The intra-assay and inter-assay coefficients of variation for sAxl and sFlt-1 were 1.7 and 4.4%; and 2.5 and 6.7%, respectively. The levels of ICAM-1 (Cat#DCD540) and endothelial nitric oxide synthase (eNOS) (Cat#DY950-05) in tissue homogenates were also detected using appropriate human ELISA kits (USCN KIT Inc., R&D). The intra-assay and inter-assay coefficients of variation for ICAM-1 were 3.7 and 6.7%, respectively. It should be noted that DuoSet ELISA kits without assay coefficients of variation were used for testing Gas6 and eNOS. Each sample was quantified twice, and the results expressed as a mean.

### Cell Viability Assays

The proliferative activity of cells was tested by water-soluble tetrazolium salt (WST-1) assays (Beyotime, China). HUVECs were seeded into 96-well plates at a density of 2 × 10^3^ cells/well. After 12 h, the cells were treated with recombinant sAxl at different levels and time points (8, 12, 24, 36, and 48 h), and 10 μl WST-1 was added per well. Cells were then incubated for 1 h, and the optical density of cells was then analyzed using an Infinite microplate reader (Tecan Group Ltd., Switzerland) at a wavelength of 450 nm.

### Cytotoxicity Assays

Cellular cytotoxicity was evaluated by standard lactate dehydrogenase release assays following the manufacturer’s instructions (Cytotoxicity Assays, Promega, United States). First, we seeded 96-well plates with 1 × 10^3^ cells. The plates were then incubated for 24 h with sAxl at different levels. Ten microliters of 10 × lysis buffer was added into each well to encourage the maximum release and then incubated for 45 min at 37°C. Next, 50 μl of the supernatant was transferred to a fresh plate, and 50 μl of the substrate was added. The plate was then sealed and incubated for 30 min at room temperature in the dark; 50 μl of stop solution was added to each well to terminate the reaction. The optical density was then read at 490 nm, and the results were calculated by the following formula:

%Cytotoxicity=100×(Experiment-EffectorSpontaneous-TargetSpontaneous)/(TargetMaximum-Spontaneous-TargetSpontaneous)

### Tube Formation Assay

BD Matrigel^TM^ (BD Biosciences, San Jose, CA) was thawed overnight at 4°C and diluted with an RPMI-1640 medium (1:8). Then, 24-well plates were coated with 0.3 ml of Matrigel and allowed to gelatinize at 37°C for 3 h. Then, 1 × 10^5^ of HUVEC suspension was seeded onto the Matrigel with different levels of sAxl. Tube formation was then recorded after 4 h of incubation by fluorescence microscopy. The mean length of tube formation was then calculated from three different fields.

### Migration Assay

A suspension containing 5 × 10^4^ cells was treated with 0.2 ml of recombinant sAxl in an FBS-free RPMI-1640 medium and inoculated into the upper compartment of a transwell. The bottom of the upper compartment featured a micropore membrane with a pore diameter of 8.0 μm; the lower compartment was loaded with 0.6 ml of a 10% complete medium. After 24 h, 4% paraformaldehyde was added for 15 min. The cells were then stained with 0.1% crystal violet for 5 min and washed three times with PBS. Cotton swabs were then used to wipe off the upper layer of cells. Five fields were randomly selected and photographed under an inverted microscope (×100 magnification; Olympus, United States).

### Real-Time Fluorescence Quantitative PCR

Total RNA was extracted by TRIzol-A+ reagent (Tiangen Biotech Co., Beijing, China). Reverse transcription reactions were then performed with a ReverTra Ace MMLV reverse transcriptase kit (Toyobo Co., Japan). Real-time PCR analyses were then performed using a 2 × Maxima SYBR Green qPCR Master Mix kit (Fermentas Inc., Canada). All experiments were conducted by following the manufacturer’s suggested protocols. A melting curve was obtained at the end of each run to distinguish specific and non-specific cDNA products. The data were normalized by GAPDH expression.

### Western Blotting

Western blotting was performed with a sodium dodecyl sulfate-polyacrylamide gel electrophoresis (SDS-PAGE) system. In brief, protein samples were separated by 10% SDS-PAGE. Proteins were transferred onto polyvinylidene fluoride (PVDF) membrane, and non-specific binding sites were blocked by using 5% milk. Subsequently, the membranes were incubated with primary antibodies: horseradish peroxidase-conjugated antibodies (goat anti-mouse and goat anti-rabbit) overnight at 4°C. Relative protein expression was then analyzed in a semiquantitative manner using an enhanced chemiluminescence plus reagent (Millipore, United States).

### Animals and Experimental Groupings

Adult Sprague-Dawley (SD) rats [license number: SCXK (Sichuan), 2008-24] weighing 250–300 g (males) and 230–270 g (females) were purchased from the Chengdu Dashuo Biotechnology Co., Ltd. (Sichuan, China). The Committee on the Ethics of Animal Experiments of West China Second University Hospital, Sichuan University approved all the protocols related to the animal experiments in this study, and the study was carried out following the National Institute of Health guidelines for the Care and Use of Laboratory Animals. Rats were housed in rooms at a suitable humidity and temperature with a 12:12-h light–dark cycle and mated overnight. The presence of a vaginal mucous plug the morning after mating marked day 0 of gestation (GD0).

The pregnant rats were randomly assigned to the study group on GD8 by different study groups. The experimental rats received 2 × 10^9^ PFU of an adenovirus expressing sAxl (Ad/sAxl) via tail vein injection (*n* = 6). The positive control rats were administered with Ad/sFlt-1 (*n* = 6). The negative control rats received Ad/Fc, while the blank control rats were treated with an equal volume of saline (*n* = 6 per group). Heart rate, systolic blood pressure (SBP), diastolic blood pressure (DBP), mean arterial pressure (MAP), and 24-h urine protein were measured before and during the pregnancy. The heart rate and blood pressure were tested by a non-invasive computerized tail-cuff method (BP-98A; Softron, Tokyo, Japan). Invasive blood pressure was detected on GD19 before sacrifice. Following the induction of anesthesia, the right carotid artery was surgically exposed and cannulated for invasive blood pressure monitoring. Then, the rats were sacrificed by injecting an overdose of 3% pentobarbital sodium (60 mg/kg) on GD19. Pups were removed and counted. We also measured and weighed each pup. We also weighed each placenta, and portions of the placentas obtained from different litters were fixed in 4% paraformaldehyde for morphological observations. The remaining samples were frozen at −80°C to await subsequent analysis. Placentas and kidney (*n* = 6 per group) morphology were then observed following hematoxylin-eosin (H&E) and Periodic Acid-Schiff (PAS) staining.

### Statistical Analysis

Statistical analyses were performed using SPSS (SPSS, Inc., Chicago, IL, United States). The data were analyzed using an independent two-tailed *t*-test and are given as mean ± standard deviation. One-way analysis of variance (ANOVA) was used when comparing more than two groups. The Mann–Whitney U test was used to determine the relationship between our results and clinical parameters. A *p*-value < 0.05 was considered statistically significant. All experiments were performed in triplicate.

## Results

### Subject Characteristics

The study population characteristics are shown in Table 1. No significant differences were noted with regards to maternal age, early pregnancy body mass index (BMI), and the gestation of sampling between the two groups (*P* > 0.05). The fetal growth restriction (FGR) rates of premature birth women and women with sPE are 36.8 and 33.2%, respectively. The premature birth group showed a heavier birth weight and a longer birth length than women with sPE.

**TABLE 1:** Characteristics of the study population.

**Group**	**Healthy pregnancy (*n* = 30) (preterm birth)**	**Preeclampsia with severe features (*n* = 30)**
Gestational age, y	31.8 ± 4.4	31.45 ± 5.6
Early pregnancy body mass index, kg/m^2^	24.3 ± 2.2	22.5 ± 3.3
Gestation of sampling, day	237.0 ± 17.2	242.3 ± 19.3
Gestation of delivery, day	240.2 ± 17.1	245.6 ± 16.3
Systolic blood pressure, mm Hg	113.6 ± 9.7	172.0 ± 10.4**
Diastolic blood pressure, mm Hg	73.4 ± 9.3	103.2 ± 10.7**
24-h urinary protein, g	/	4.9 ± 1.8 30 (100%)
Thrombocytopenia	1 (3.3%)	9 (30.0%)**
Impaired liver function	/	17 (56.7%)
Progressive renal insufficiency	/	15 (50.0%)
Pulmonary edema	/	6 (20.0%)
New-onset cerebral or visual disturbances	/	2 (6.7%)
Birth weight, g	1966.8 ± 528.8	2164.0 ± 886.0
Birth length, cm	47.8 ± 2.8	45.3 ± 4.5
Fetal growth restriction rate	36.8%	33.2%

Thrombocytopenia: platelet count less than 100,000/microliter; impaired liver function: abnormally elevated blood concentrations of liver enzymes (to twice normal concentration), severe persistent right upper quadrant or epigastric pain unresponsive to medication and not accounted for by alternative diagnoses, or both; progressive renal insufficiency: serum creatinine concentration greater than 1.1 mg/dL or a doubling of the serum creatinine concentration in the absence of other renal disease. The results are presented as the mean ± SD.

** Represents pregnant women with preeclampsia and severe features (sPE) or pre-term pregnancy group compared with term pregnancy group, ** P < 0.01.

### The Plasma Levels of sAxl and Key Angiogenic Molecules in sPE

**FIGURE 1:**
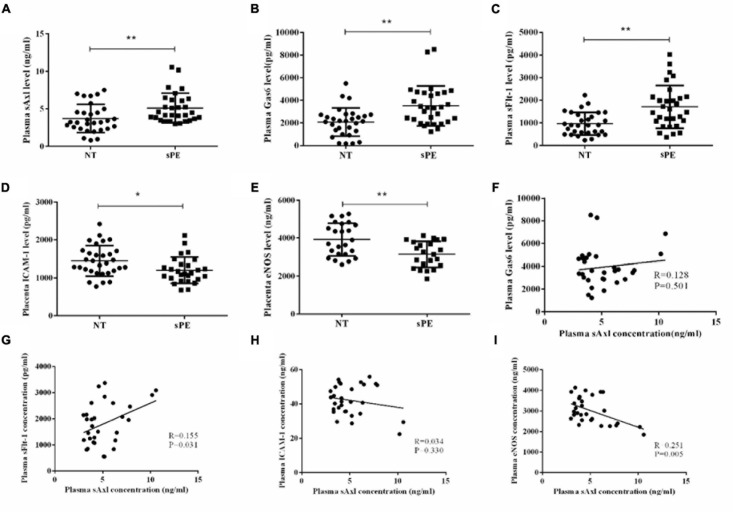
The level of plasma sAxl and key molecules of angiogenesis in preeclampsia with severe features (sPE). **(A–C)** Plasma sAxl, Gas6, and sFlt-1 concentration were measured by ELISA in normal pregnants (NT) and sPE. **(D,E)** The ICAM-1 and eNOS levels in the homogenate of placental tissue were detected in women with NT and sPE. **(F–I)** Correlations between plasma sAxl and plasma Gas6, sFlt-1 and placental ICAM-1, eNOS. The results are presented as mean ± SD. *p < 0.05, **p < 0.01 *vs.* respective controls. *n* = 30 per group.

ELISA was used to evaluate the plasma levels of sAxl, Gas6, and sFlt-1, and the expression levels of ICAM-1 and eNOS in placentas. The maternal plasma levels of sAxl increased in the third trimester. Women with sPE exhibited significantly higher plasma levels of sAxl, Gas6, and sFlt-1 than pregnant women who did not have PE (*p* < 0.01, *p* < 0.05, Figures 1A–C). Furthermore, the placental levels of ICAM-1, and eNOS, were significantly lower in women with sPE (*p* < 0.05, Figures 1D,E). Besides, the plasma levels of sAxl were positively correlated with the plasma levels of sFlt-1 and negatively correlated with the placental levels of eNOS (*p* < 0.05, Figures 1G,I). However, there was no significant correlation between plasma levels of sAXL and ICAM-1 or GAS6 (Figures 1F,H). These data implied that sAxl might participate in the pathogenesis of sPE by affecting the function of endothelial cells.

### Correlation Analysis of Plasma sAxl Level and Clinical Indicators in sPE

**FIGURE 2:**
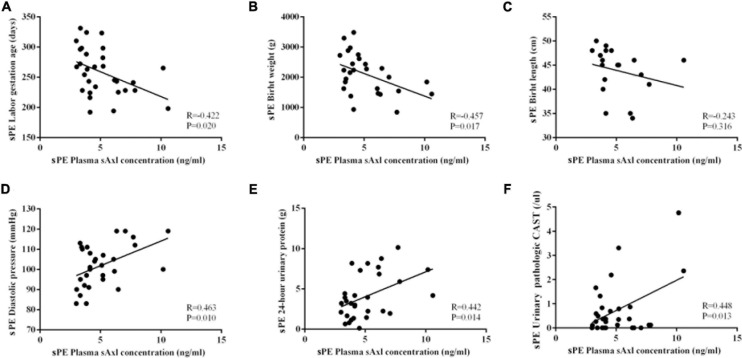
Correlation analysis of the plasma sAxl level and clinical indicators in preeclampsia with severe features (sPE). **(A)** Gestational age. **(B,C)** Birth weight and length. **(D)** Diastolic blood pressure. **(E)** 24-h urinary protein. **(F)** Urine pathologic CAST. *n* = 30 per group.

Next, we further analyzed the correlation between the plasma levels of sAxl and the clinical indicators of pregnant women with sPE. The elevated levels of sAxl were negatively correlated with the gestational age, birth weight, and birth length (Figures 2A–C), but positively correlated with blood pressure, 24-h urine protein, and urine pathological CAST (Figures 2D–F).

### Effects of sAxl on HUVECs Proliferative, Tube Formation, Migration, and Functions and the Molecular Mechanisms Associated With This Effect

**FIGURE 3:**
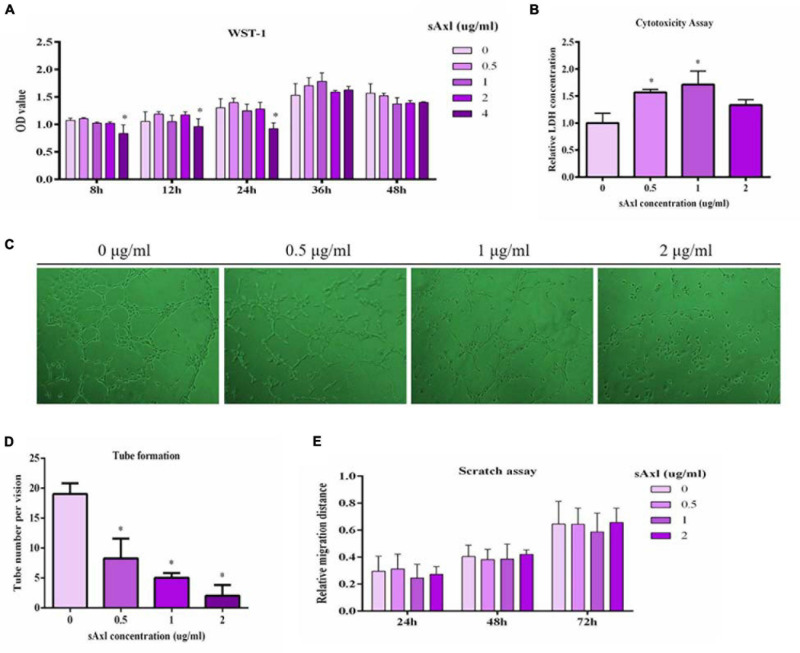
Effects of sAxl on endothelial proliferative, cytotoxicity, tube formation, and migration. **(A)** Cell proliferation was detected by WST-1. **(B)** Cytotoxicity assay was evaluated by the LDH release assay. **(C,D)** The tube formation of vascular endothelial cells after 8 h of sAxl culture at different levels was observed. **(E)** Migration ability was detected by scratch assay. The results are presented as mean ± SD. *p < 0.05, **p < 0.01 vs. respective controls. *n* = 3 per group.

Cell proliferation was estimated by a WST-1 test. We found that recombinant human sAxl (4 μg/ml) significantly inhibited the proliferative ability of endothelial cells (*p* < 0.05, Figure 3A). Furthermore, sAxl (0–2 μg/ml) destroyed the integrity of the endothelial cell membranes in a dose-dependent manner, leading to a significantly increased LDH level in the medium (*p* < 0.05); however, there was no significant effect with the 2 μg/ml sAxl treatment (Figure 3B). We also found that recombinant human sAxl significantly reduced the ability of vascular endothelial tube formation. When the level of sAxl was further increased to 2 μg/ml, vascular endothelial cells almost lost the ability to tube formation (*p* < 0.05, Figure 3C). Moreover, the number of vascular endothelial cells was significantly reduced in a dose-dependent manner (*p* < 0.05, Figure 3D). However, sAxl had no significant effect on migration (Figure 3E).

**FIGURE 4:**
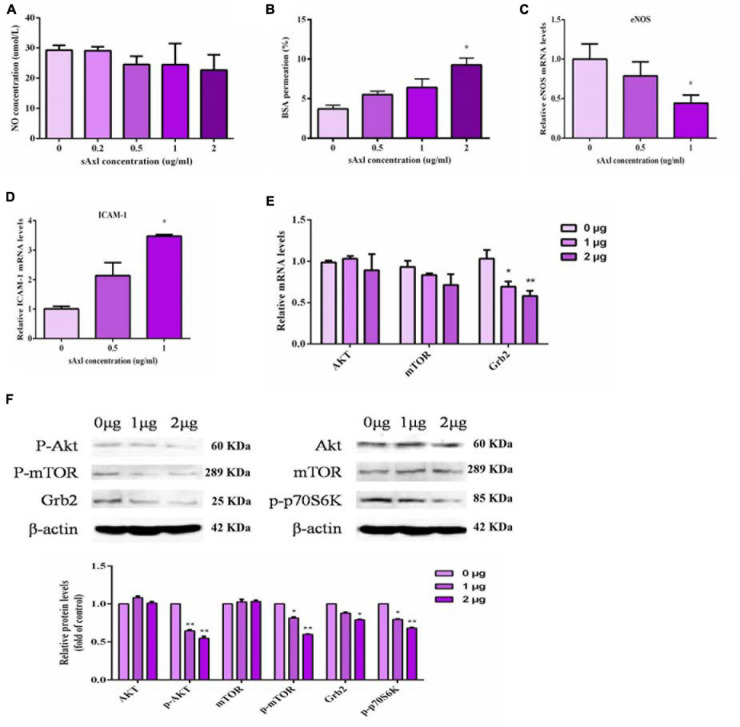
Effects of sAxl on endothelial NO, eNOS, and ICAM-1. **(A)** NO level in medium supernatant after treatment with different sAxl levels. **(B)** BSA permeation. **(C,D)** mRNA expression of eNOS and ICAM-1. **(E,F)** The expression of signal molecules at different levels of sAxl at different times. The results are presented as mean ± SD. *p < 0.05, **p < 0.01 *vs.* respective controls. *n* = 3 per group.

Next, we measured the levels of NO in the culture medium, the BSA permeation, and a range of factors related to endothelial function. Human recombinant sAxl had no obvious effect on the NO level after treatment with recombinant human sAxl (Figure 4A). Recombinant human sAxl protein increased the fluoresce-labeled BSA permeation and weakened the function of the endothelial cell barrier. When the level of sAxl in the medium reached 2 μg/ml, the barrier function of vascular endothelial cells was significantly impaired, and the BSA permeation was significantly higher than that of the untreated group and the group treated with a low level of sAxl (*p* < 0.05, Figure 4B). We also found that recombinant human sAxl induced a reduction in the expression of eNOS mRNA in HUVECs, but significantly increased the expression of ICAM-1 (*p* < 0.05, Figures 4C,D).

Our results also showed that recombinant sAxl could significantly downregulate the expression of p-Akt, p-p70S6K, p-mTOR, and Grb2 (*p* < 0.05, *p* < 0.01, Figures 4E,F) but did not affect the expression of either Akt and mTOR. These indicated that sAxl inhibited the activation of the transmembrane receptor protein Axl by competitively binding to Gas6, resulting in the inhibited m-phosphorylation of mTOR and p-p70S6K, thus reducing the conversion of Akt to p-Akt. In turn, this caused the inhibition of mTOR phosphorylation and p-p70S6K expression. The expression of Grb2 was also reduced, eventually causing endothelial dysfunction.

### Establishment of a Rat Model With High Levels of sAxl

#### Heart Rate, Blood Pressure, and Urinary Protein

**FIGURE 5:**
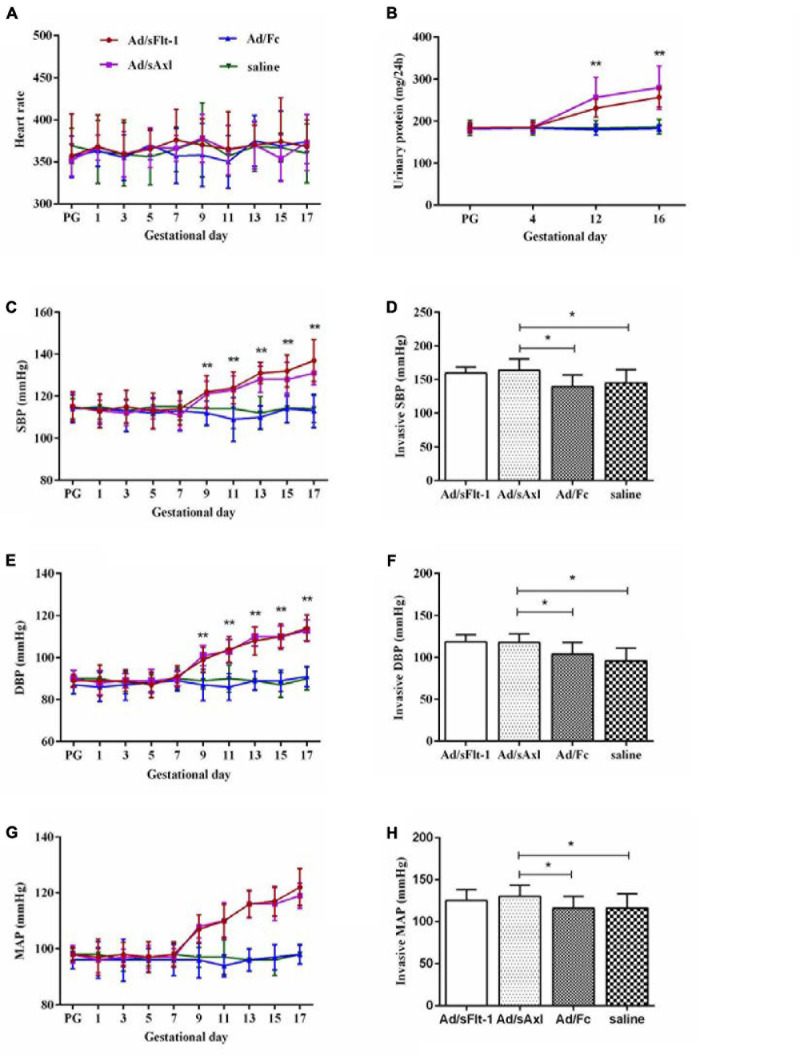
Effects of sAxl on preeclampsia-like symptoms of pregnant rats. **(A,C,E,G)** Heart rate, systolic blood pressure (SBP), diastolic blood pressure (DBP), and mean arterial pressure (MAP) were measured at baseline and from the first day to the 17th day of pregnancy in four groups. **(B)** Urine protein level. **(D,F,H)** Invasive SBP, invasive DBP, and invasive MAP. The results are presented as mean ± SD. *p < 0.05, **p < 0.01 vs. respective controls. *n* = 6 per group.

We measured the heart rate and the blood pressure to determine whether sAxl induced hypertension in rats. Pregnant rats treated with sAxl showed no significant changes in the heart rate; however, SBP, DBP, and MAP were all significantly increased from GD9 to GD17 (*p* < 0.01, Figures 5A,C,E,G). We also observed that invasive SBP, invasive DBP, and invasive MAP were all significantly increased on GD19 (*p* < 0.05, Figures 5D,F,H). The results of blood HGB, PLT counts, and biochemical index values are shown in Table 2. Compared to the saline and Ad/Fc groups, there were no significant changes with regards to blood HGB and PLT. Furthermore, the levels of ALT, AST, LDH, and Cr were increased significantly (*p* < 0.05). These findings suggested that sAxl was capable of damaging liver function in rats.

**TABLE 2:** Blood HGB, PLT counts and biochemical index values (*n* = 6, $\bar{\text{X}}$ ± SD).

**Groups**	**Ad/sFlt-1**	**Ad/Axl**	**Ad/Fc**	**Saline**	***F*-value**	***P-*value**
HGB (g/L)	9811.6	11011.3	10710.0	11119.1	1.165	0.348
PLT (×10^9^/L)	29389.9	360184.0	419178.0	413157.0	0.829	0.493
ALT (U/L)	6413.3*	6712.1*	437.6	5210.2	6.471	0.003
AST (U/L)	23672.2**	238110.0**	10834.6	11615.0	6.928^◄^	0.009
LDH (U/L)	1840652.9**	2309845.0**	585450.5	621147.9	13.340	<0.001
Cr (μmol/L)	609.1*	6416.3*	425.5	455.4	6.935	0.002
UA (mmol/L)	4.80.9	5.72.2	4.51.2	5.11.9	0.625	0.607

The results are presented as the mean ± SD. ^◄^ represents the F value after Welch correction when the variances are not homogeneous. * P < 0.05, ** P < 0.01 vs. control (Ad/Fc and saline).

Further analysis showed that the urinary protein levels of the Ad/sFlt-1 positive control group and the Ad/sAxl experimental group were higher than those of the Ad/Fc negative control group and the saline control group on GD12 and GD16 (*p* < 0.01). The urinary protein level of pregnant rats in the Ad/sAxl experimental group was 186 ± 12.4 mg/day on GD4 and increased to 280 ± 51.4 mg/day on GD16 after treatment. The urinary protein level of pregnant rats in the Ad/sFlt-1 positive control group was 186 ± 10.1 mg/day on GD4 and increased to 257 ± 23.6 mg/day by GD16 (Figure 5B).

#### Maternal Placenta, Kidney, and Fetal Development

**FIGURE 6:**
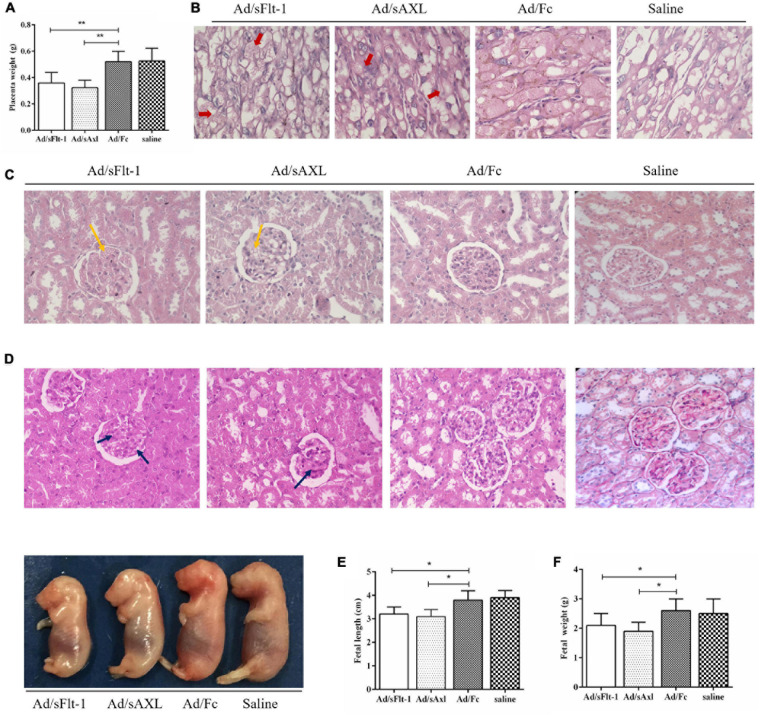
Effects of sAxl on the maternal placenta, kidney, and fetal development in rats. **(A)** Placenta weight. **(B)** Placenta morphology was detected by HE staining (400x, red arrow). **(C,D)** Kidney morphology was detected by H&E and PAS staining (400x, yellow and blue arrow). **(E,F)** The fetal weight and length of rats. The results are presented as mean ± SD.*p < 0.05, **p < 0.01 vs. respective controls. *n* = 6 per group.

Placental weight was decreased significantly in the Ad/sFlt-1 positive control and the Ad/sAxl groups (*p* < 0.01, Figure 6A). H&E staining showed that the placenta villi were swollen in the Ad/sFlt-1 group and the Ad/sAxl group (red arrow, Figure 6B). Both these groups presented with an increased number of mesangial cells and slightly swollen glomeruli (yellow arrow), and no significant changes were observed in the endothelial cells (Figure 6C). Furthermore, PAS staining showed mild swelling in the renal glomeruli of Ad/sAxl and Ad/sFlt-1 rats, along with mesangial hyperplasia and swollen endothelial cells (blue arrow, Figure 6D). The lengths of the fetal rats were significantly shorter in the Ad/sFlt-1 and Ad/sAxl groups (3.5 ± 0.3 and 3.6 ± 0.3 cm, respectively) than in the Ad/Fc negative control (3.8 ± 0.4 cm) and the saline blank control groups (3.9 ± 0.3 cm; *p* < 0.05, Figure 6E). The fetal weight in the Ad/sFlt-1 and Ad/sAxl groups were significantly reduced (*p* < 0.05, Figure 6F).

#### sAxl Affected the Function of Vascular Endothelial Cells *in vivo*

**FIGURE 7:**
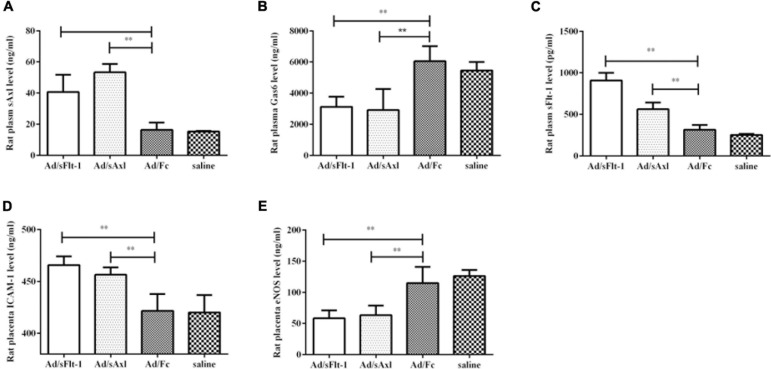
Effects of sAxl on rat vascular endothelial sAxl, Gas6, sFlt-1, and adhesion molecules. **(A–C)** The expression of sAxl, Gas6, and sFlt-1 in plasma. **(D,E)** ICAM-1 and eNOS expression levels in the placenta. The results are presented as mean ± SD. **p < 0.01 vs. respective controls. *n* = 6 per group.

The levels of plasma sAxl in the Ad/sFlt1 and Ad/sAxl groups were significantly higher than those in the Ad/Fc negative control and the saline control groups (*p* < 0.01, Figure 7A). The plasma levels of Gas6 in the Ad/sAxl and Ad/sFlt-1 groups had decreased significantly (*p* < 0.01, Figure 7B). The plasma levels of sFlt-1 in the Ad/sFlt-1 and Ad/sAxl groups were significantly higher than those in the Ad/Fc negative control and the saline control groups (*p* < 0.01, Figure 7C). The placenta levels of ICAM-1 in the Ad/sFlt-1 and Ad/sAxl groups were significantly higher than those in the Ad/Fc and saline groups (*p* < 0.01, Figure 7D). In contrast, the levels of eNOS were significantly reduced in the Ad/sFlt-1 and Ad/sAxl groups (*p* < 0.01, Figure 7E).

## Discussion

PE is a systemic vascular disorder that is characterized by new-onset hypertension after 20 weeks of gestation. This condition is the leading cause of maternal and perinatal morbidity and mortality. However, it is still unclear what mechanisms are responsible for endothelial dysfunction in PE. Our previous study shows that the plasma levels of sAxl in sPE were markedly higher than those of pregnant women without PE; these results suggest that sAxl may be involved in sPE (Liu et al., 2014). In the present study, we verified our previous findings and further investigated the possible mechanisms of sPE induced by an increased level of sAxl.

Membrane-bound Axl consists of the extracellular region of the protein; this is shed from the cell membrane as a result of proteolysis and circulates in the blood in a soluble form (sAxl) (O’Bryan et al., 1995; Costa et al., 1996). In this study, we found that the elevated levels of sAxl were negatively correlated with gestational age, along with birth weight and length, but were positively correlated with blood pressure, 24-h urinary protein level, and urine pathological CAST. These data implied that sAxl might participate in the pathogenesis of sPE by affecting the function of endothelial cells. To prove this hypothesis, we carried out experiments in HUVECs and found that recombinant human sAxl (4 μg/ml) exerted certain inhibitory effects on the proliferative ability of endothelial cells and that sAxl (0–2 μg/ml) could destroy the integrity of the endothelial cell membranes in a dose-dependent manner. This treatment also reduced the ability of vascular endothelial cells to form tubes. We established an adenovirus sFlt-1 (ad/sFlt-1), PE-like mouse model, as previously described, to act as a positive control (Venditti et al., 2014). We found that Ad/sAxl and Ad/sFlt-1 groups exhibited increased levels of plasma sAxl and sFlt-1, respectively, and presented with a PE-like phenotype. In the Ad/sAxl and Ad/sFlt-1 rats, we observed an increase in SBP, DBP, and MAP, from GD9 to GD17. Furthermore, blood HGB and PLT, along with the levels of ALT, AST, LDH, and Cr, were significantly increased. Urinary protein levels were higher on GD12 and GD16, and the maternal placenta showed abnormal morphology. Moreover, the fetal length and weight of rats were reduced in ad/sAxl and ad/sFlt-1 rats; thus, ad/sAxl offspring may have experienced FGR. These data suggest that higher sAxl levels could induce sPE by destroying the function of endothelial cells.

Gas6 and sAxl are present in the circulatory systems of both mice and humans and circulate together in a high-affinity complex (Ekman et al., 2010). When circulating, Gas6 is bound to sAxl; this suggests that circulating Gas6 is inhibited when the levels of sAxl increase, thus becoming incapable of stimulating TAM receptors, such as Axl (Ekman et al., 2010). The Gas6/TAM system has been implicated in cell proliferation, cell adhesion and migration, phagocytosis, and the release of inflammatory cytokines (Manfioletti et al., 1993; Godowski et al., 1995). Moreover, Axl is known to be involved in the integrity of the vasculature; a study indicated a role for Gas6/Axl signaling in promoting the angiogenic potential of renal cell carcinoma cells (Xiao et al., 2019). We found that the plasma levels of sAxl and Gas6 in women with sPE were significantly higher than those in pregnant women who did not have PE. Moreover, in HUVECs, sAxl was shown to significantly downregulate the expression levels of p-Akt, p-p70S6K, p-mTOR, and Grb2 but had no significant effect on Akt and mTOR. Thus, sAxl may inhibit the activation of the transmembrane receptor protein Axl by competitively binding to Gas6 (in an autocrine or paracrine manner) in the culture medium. This inhibited the m-phosphorylation of TOR and the expression of p-p70S6K, thus reducing the conversion of Akt to p-Akt. The expression of Grb2 decreased, eventually causing vascular endothelial cell dysfunction. The plasma levels of Gas6 in pregnant women with sPE were elevated, but the plasma levels of Gas6 in the Ad/sAxl group and the Ad/sFlt-1 group were decreased. Peng et al. (2018) found that elevated expression of the plasma levels of sAxl, as well as the reduction of Gas6, was observed in sPE. However, the maternal Gas6 serum levels are significantly increased in PE during pregnancy (Stepan et al., 2013). Gas6/Axl signaling is capable of inducing PE in pregnant rats (Hirschi et al., 2020). Therefore, the expression of Gas6 in PE is controversial. In this study, experimental studies were carried out in combination with placenta, cells, and animals; it was found that the decreased level of Gas6 could induce sPE. We speculated that Gas6 may activate downstream signaling but cannot because it is sequestered by sAxl perhaps in sPE. Our findings indicated that higher levels of sAxl induced sPE *via* the Gas/Axl pathway.

Failure of the uterine spiral artery remodeling results in reduced placental perfusion; subsequently, placental factors are released into the maternal circulation that can lead to the dysfunctional activity of the endothelial cells (Salomon et al., 2014). VEGF and PlGF have both been shown to promote endothelial cell function (Neagoe et al., 2005). VEGF is known to mediate vasodilation *via* both VEGFR1 (Flt1) and VEGFR2 receptors; sFlt1 is known to interact with both VEGF and PlGF. The release of placental-derived antiangiogenic factors (such as sFlt-1) into the maternal circulation can cause an imbalance in angiogenic factors (VEGF and PLGF), thus leading to endothelial dysfunction and PE symptoms (Cindrova-Davies, 2014). Compared to pregnant women who do not have PE, the circulating levels of the antiangiogenic factor sFlt-1 and the expression levels of placental sFlt-1 mRNA are known to be higher in women with PE (Quezada et al., 2020).

Also, eNOS is a key enzyme for NO production and plays an important role in the regulation of blood pressure via its effect on vasodilation. A previous study reports that increased levels of sFlt-1 can induce a PE-like syndrome in rats, linked with increased levels of endothelin 1 and reduced levels of NO, thus resulting in endothelial dysfunction (Murphy et al., 2010). In addition, many endothelium-derived factors, and markers of endothelial function, are known to be aberrantly expressed in PE, including ICAM-1, eNOS, and sVCAM-1 (Tuzcu et al., 2015; Wang et al., 2015). In the present study, we found that plasma levels of sFlt-1 were reduced in women with PE and that placental levels of ICAM-1 and eNOS levels were also markedly lower; these findings concur with previous studies (Cindrova-Davies et al., 2011). The plasma concentration of sAxl was positively correlated with plasma sFlt-1 concentration and negatively correlated with the placental concentration of eNOS. Moreover, we found that Ad/sAxl-rats had increased levels of ICAM-1 and sFlt-1 but decreased levels of eNOS. We also observed that the Ad/sFlt-rats exhibited increased plasma levels of sFlt-1 and placental levels of ICAM-1, and reduced levels of eNOS; however, plasma sAxl did not change significantly. Therefore, this study suggested that elevated sAxl levels may induce an increase in the levels of sFlt-1; but it remains unclear whether there is a direct interaction between sAxl and sFlt-1. It is also worth noting that levels of ICAM-1 were decreased in sPE compared with pregnant women who did not have PE. This may be related to the current methods used to treat sPE, such as the administration of magnesium sulfate. Taken together, these data indicate that sAxl might play a role in the function of endothelial cells by affecting the levels of vascular endothelial factors.

## Conclusion

In conclusion, for the first time to our knowledge, this study showed that increased levels of sAxl had cytotoxicity effects on endothelial cells. Furthermore, we demonstrated that an increased plasma level of sAxl might induce placental endothelial dysfunction and induce sPE *via* the regulation of the Gas6/Axl pathways and the levels of certain angiogenic molecules, including ICAM-1, eNOS, and sFlt-1. In addition, we established a novel rat model that expressed high levels of sAxl by injecting an adenovirus that overexpressed sAxl. The model rats exhibited placental endothelial dysfunction and a PE-like phenotype. Many previous studies have shown that Gas6 can induce alterations in the expression of certain tissue factors in endothelial cells. Thus, we speculate that sAxl might induce placental endothelial dysfunction through the regulation of key angiogenic molecules via the Gas6/Axl pathways. Future studies should investigate these mechanisms further. This study suggests that sAxl could be a potential biomarker and therapeutic agent for sPE.

## Data Availability Statement

The original contributions presented in the study are included in the article/supplementary material, further inquiries can be directed to the corresponding author/s.

## Ethics Statement

The studies involving human participants were reviewed and approved by the Medical Ethics Committee of Sichuan University. The patients/participants provided their written informed consent to participate in this study. The animal study was reviewed and approved by the Committee on the Ethics of Animal Experiments of West China Second University Hospital, Sichuan University. Written informed consent was obtained from the individual(s) for the publication of any potentially identifiable images or data included in this article.

## Author Contributions

SG and SZ: data curation, investigation, and writing the original draft. ML: investigation and software operation. YZ: data curation and investigation. LG and TW: investigation and project administration (supporting). RZ: funding acquisition, project administration, and writing and editing. All authors read and approved the final version of the manuscript.

## Conflict of Interest

The authors declare that the research was conducted in the absence of any commercial or financial relationships that could be construed as a potential conflict of interest.
